# Supplementary material for: Serum KL-6 as a Candidate Predictor of Outcome in Patients with SARS-CoV-2 Pneumonia
Source: J Clin Med. 2023 Oct 26;12(21):6772. doi: 10.3390/jcm12216772 (PMC10648641; doi:10.3390/jcm12216772)
Supplement: Supplementary file 1 [file jcm-12-06772-s001.zip › jcm-2651797-supplementary.pdf]

Supplemental Material

Table S1: Inclusion and Exclusion criteria of the present study.

**Inclusion criteria**	**Exclusion criteria**
• Patient with SARS-CoV-2 pneumonia* independent of preexisting lung diseases or other comorbidities<br><br>• Written informed consent	• Age < 18 years<br>• Pneumonia due to other causes than SARS-CoV-2<br><br>• Inability to provide informed consent due to the clinical condition<br>• Contraindications to blood sampling
* according to C. Huang et al. 2020 [2]	

Table S2: WHO Clinical Progression Scale of COVID-19 modified presentation of J. C. Marshall et al. 2020 [22].

**Score**	**Details**	**Patient scale**
0-3	Ambulatory	Mild disease
4	No oxygen	Moderate Disease:
5	Oxygen by mask or nasal prongs	Hospitalized
6	Non invasive ventilation or high flow	Severe Disease:
7	Intubation and mechanical ventilation paO2/FiO2 ≥ 150	Hospitalized, ICU
8	Mechanical ventilation paO2/FiO2 ≤ 150 or vasopressin	
9	Mechanical ventilation paO2/FiO2 ≤ 150, dialysis or ECMO	
10	Death	Dead

Table S3: Serum KL-6 values at baseline according to WHO Clinical Progression Scale [22].

**WHO Score**	**4**	**5**	**6**	**7-9**	**10**
N	21	68	48	5	15
**KL-6**, U/ml	364<br>[230 - 517]	358<br>[246 - 389]	515<br>[329 - 753]	535<br>[387 - 1021]	608<br>[348 - 935]
